# Green public facilities and residents’ health from a socio-spatial perspective

**DOI:** 10.3389/fpubh.2026.1972132

**Published:** 2026-10-30

**Authors:** Zeyu Zhang, Yuanchao Wang

**Affiliations:** School of Philosophy and Social Development, Shandong University, Jinan, China

**Keywords:** green public facilities, occupational status, public transport provision, self-rated health, spatial balance

## Abstract

**Background:**

This study conceptualizes green public facilities as green-space resources embedded in urban public-resource allocation and examines the relationship between their spatial distribution and residents’ health from a socio-spatial perspective.

**Methods:**

Data on 6,871 respondents from the 2021 Chinese General Social Survey were linked to green public facility and public transport point-of-interest data and statistical indicators for 82 districts/counties. Weighted ordinary least squares models were estimated with district/county-clustered robust standard errors.

**Results:**

Greater spatial balance of green public facilities was positively associated with self-rated health in some models, but the associations were small and sensitive to model specification. The association was stronger among respondents with lower occupational status, and this interaction was the most robust finding. Better public transport connectivity was associated with a weaker relationship between spatial balance and health, but this interaction was more sensitive to alternative indicators and the treatment of districts/counties with no recorded green facilities.

**Discussion:**

These findings suggest that the health relevance of green public facilities should be considered in relation to their provision and spatial distribution, social groups’ capacity to use these resources, and public transport connectivity. Evidence for the occupational-status interaction was more consistent than evidence for the overall association or public transport moderation.

## 1 Introduction

Health is a fundamental human need and component of wellbeing. From a sociological perspective, health is not merely a medical outcome but is deeply embedded in social structures. At the individual level, education, occupation, income, and housing shape people’s capacity to respond to health risks and obtain health resources (1, 2). At the macro level, social security, public services, and living environments are important conditions for residents’ health. In addition to conventional medical resources such as hospitals and clinics, green public facilities are also closely related to health.

The allocation of green public facilities is a shared concern in the sociology of health and urban environmental research. Parks and public green spaces provide settings for recreation, psychological restoration, and social interaction, while their health relevance also depends on residents’ social circumstances. Understanding this relationship therefore requires attention to both facility allocation and the conditions under which different groups can use these resources.

Green public facilities refer to outdoor recreational spaces in cities that are open to the public, such as parks, public green spaces, and greenways. They combine recreational and environmental-regulation functions and form part of the broader green environment, while remaining distinct from the overall distribution of forests, wetlands, and other natural vegetation. These facilities are directly embedded in residents’ everyday lives. A large body of research has identified potential health benefits through reduced environmental risks, increased physical activity and social interaction, and relief from psychological stress (3–5).

The total supply of green public facilities does not fully describe their distribution within local areas. Districts/counties with similar numbers of facilities may differ in whether those facilities are concentrated in a few subdistricts/towns or spread across many. Building on research on social differences in green-space accessibility, this study examines facility allocation among subdistricts/towns within districts/counties. Spatial balance is defined as the evenness of facility point-of-interest (POI) counts across these subdistricts/towns. This indicator describes the resource-allocation context in which residents live. Its relationship with opportunities to use facilities also depends on population distribution, facility size, travel distance, and opening conditions. It therefore cannot be equated with actual accessibility or equity in resource use. Examining spatial balance and health can complement research on total provision and individual accessibility and contribute to understanding the spatial basis of health differences.

Socio-spatial theory provides a basis for understanding this relationship. From this perspective, urban space is socially produced (6, 7). Likewise, the spatial distribution of green public facilities is both a product of social structure and an important part of its reproduction. The health relevance of green public facilities therefore cannot be explained solely by the amount provided. It is also necessary to ask where these facilities are located, who can reach them more easily, who is more likely to use them, and which residents can convert these spatial resources into health benefits.

This study examines the relationship between the spatial distribution of green public facilities and residents’ health, bringing a socio-spatial perspective to research on public resources and health. It addresses three empirical questions. First, is the spatial distribution of urban green public facilities associated with residents’ health? Second, does this association vary by social position? Third, does public transport moderate this association?

The relationship between green public facilities and residents’ health is an important topic in urban environmental research, public health, and the sociology of health. Existing studies have found that parks, green spaces, and greenways are relevant to both physical and mental health (3–5, 8). Three broad mechanisms have been proposed: environmental risk reduction, psychological restoration, and everyday behavior.

First, environmental risk reduction may occur when parks, green spaces, and greenways improve the urban microenvironment. They can help reduce air pollution and noise, mitigate the urban heat-island effect, and regulate the local climate (3, 9, 10). Green public facilities can thus provide outdoor environments with lower pollution and noise levels that are suitable for spending time outdoors, potentially reducing the adverse health consequences of everyday environmental stressors.

Second, green public facilities may support psychological restoration through stress relief, attention restoration, and emotional regulation. Commuting pressure, work stress, residential crowding, and environmental stimuli can contribute to mental fatigue and emotional tension. Natural landscapes, open spaces, and green environments provide opportunities for rest and relaxation and allow residents to step away temporarily from the demands of urban life (9, 11, 12). Their potential health benefits therefore extend beyond disease risk to perceived stress, emotional wellbeing, and everyday experience.

Third, green public facilities may influence health through physical activity and social interaction. They provide convenient spaces for leisure, sports, and exercise and can help increase physical activity (13, 14). They are also public spaces where residents meet, spend time, and communicate, potentially strengthening neighborhood interaction, social support, and a sense of community. Dadvand et al. (15) examined mental health, social support, and physical activity together in relation to green space and general health, highlighting the need to consider these processes jointly. Green public facilities therefore serve as settings for everyday life and social interaction as well as environmental resources.

Despite the large body of empirical research on green public facilities and health, findings are not consistent. Associations vary across cities, population groups, and measurement approaches, and may be absent or reversed in some contexts (5, 16). For example, using Dutch cohort data, Picavet et al. (17) found that longer residence in a greener environment or increases in surrounding greenness did not correspond to clearer health advantages. The health relevance of green space therefore needs to be evaluated in relation to specific settings and health outcomes. After grading the evidence from existing meta-analyses, Xie et al. (18) reported that associations between green space and health generally favored beneficial outcomes, but stronger evidence remained limited to a small set of health outcomes. Differences in study design, bias, and precision also need to inform the interpretation of this literature.

Disagreements among empirical studies may arise partly from differences in indicators and spatial scales. Studies use measures such as green-space area, park counts, vegetation coverage, perceived green space, and visible greenery in street-view images. These capture distinct dimensions, including resource quantity, vegetation cover, and subjective experience (19–22). Jarvis et al. (22) found only a weak correlation between public green-space accessibility and natural land cover around residences, indicating that these measures are not interchangeable. A systematic review by Nguyen et al. (23) also found that relationships with health differed by green-space characteristics, including size, vegetation type, and connectivity, which cannot be captured adequately by a single count measure. The definition of geographic context can also influence conclusions about environmental conditions and health (24, 25). Green public facilities can be measured at the levels of prefecture-level cities, districts/counties, subdistricts, communities, or residential neighborhoods (20, 25). Higher-level units may obscure heterogeneity among districts/counties, while residential-neighborhood measures may underestimate activity spaces because people also encounter green spaces at workplaces, along commuting routes, and in places used for leisure or shopping (26–28).

These measurement differences complicate comparisons across studies, while three theoretical issues warrant further investigation. First, facility provision and spatial distribution need to be examined together. Research on public services has addressed spatial layout, accessibility, and social equity and emphasized evaluating facility allocation in relation to residents’ needs (29). When green facilities are concentrated in a few areas, conditions of use may differ across neighborhoods even where total provision is high. Ignoring spatial distribution can therefore obscure both health relationships and potential spatial inequalities. Previous studies have examined inequalities in park allocation across social groups and methods for measuring accessibility (30, 31). In Shenzhen, Huang et al. (32) found that differences in park accessibility across housing groups varied by park type and transport mode. Comparing three US urban areas, Heo and Bell (33) similarly found that relationships between sociodemographic characteristics and green space depended on the measure used; better access to park entrances did not necessarily correspond to higher values of other greenness indicators. This work forms part of a broader literature on spatial equity in green public facilities (19, 31, 34). Building on these studies, further research is needed on the relationship between the distribution of facilities across subdistricts/towns within districts/counties and residents’ health. The present study concerns the allocation context represented by facility counts, whose evenness cannot be equated with actual accessibility or equity in use.

Second, the role of social stratification requires further examination. Although green public facilities are open to all residents, social groups differ in their capacity to reach and use these resources because of differences in housing options, discretionary time, and transport. Mitchell and Popham (35) found smaller income-related inequalities in all-cause and circulatory-disease mortality in greener areas of England. A review by Rigolon et al. (36) also suggested that associations between green space and health were often stronger among groups with lower socioeconomic status, although this pattern varied by green-space type and geographic context. However, Pearson et al. (37) argued in a critical discussion that disadvantaged groups do not necessarily obtain greater health benefits and that such conclusions depend on how disadvantage is defined, study design, and interpretation. These studies provide a basis for examining group differences, but whether occupational status has a similar moderating role requires empirical testing. Situating green-space distribution and its health relationships within social inequality is therefore necessary to understand the production and reproduction of social space.

Third, relationships between green public facilities and other features of the urban environment deserve more attention. The potential benefits of green public facilities depend in part on other spatial conditions, particularly public transport. Residents’ environmental exposure extends beyond their homes and is shaped by their activity spaces, which public transport can help expand (38, 39). In Hong Kong, Tao et al. (40) found that associations of bus and rail access with activity spaces and participation in non-work activities varied by income and commuting status. Transport conditions therefore do not operate uniformly across groups. In research on green public facilities and health, public transport is often treated as a background feature of the built environment. Whether it moderates the relationship between facility distribution and health remains insufficiently understood.

A socio-spatial perspective can help address these questions by examining how green public facilities are distributed across urban space, how this distribution relates to health, whether the relationship varies across social groups, and whether public transport moderates it.

This study understands the relationship between green public facilities and residents’ health as socio-spatially structured rather than as an automatic consequence of facility provision. It emphasizes three dimensions. The first is the distribution of spatial resources, namely whether green public facilities are evenly distributed within cities. The second is the production of space, namely whether social stratification shapes residents’ capacity to obtain potential health benefits from green-space resources. The third is spatial accessibility, namely whether public transport changes residents’ dependence on the balanced local distribution of green facilities.

Since the spatial turn in the social sciences in the 1970s, scholars have increasingly recognized the social significance of space beyond its material form and geographic coordinates. As Lefebvre argued, space is a product of social relations and, in turn, contributes to their production (6, 7).

The spatial distribution of green public facilities is likewise closely related to social structure. These resources are often concentrated in particular parts of a city, producing uneven patterns of provision. Although the facilities are open to all residents, people living farther from concentrations of green resources may face greater time and travel costs. Total facility supply may therefore have a limited relationship with average health if opportunities to use these resources are concentrated among a subset of residents. At a given level of provision, a more balanced distribution may broaden opportunities for environmental risk reduction, psychological restoration, physical activity, and social interaction. This expectation concerns the potential relevance of the allocation context; it does not imply that equal facility counts necessarily provide equal access.

*Hypothesis 1*: Greater spatial balance of urban green public facilities is associated with better health among residents.

The spatial distribution of green public facilities is embedded in social stratification and may contribute to the reproduction of social structure. Social-status groups differ in residential location, time resources, mobility, and access to alternatives (30, 41, 42). Advantaged groups can more readily afford high-quality housing near green spaces and may have greater influence over local environmental conditions and planning decisions. Collective action, including opposition to undesirable local developments, can reinforce these advantages. Such processes may bring affluent residential areas into closer proximity to green public facilities while leaving disadvantaged groups with fewer opportunities to use them.

Advantaged groups may also have greater flexibility in their use of time and access to convenient transport, including private cars, allowing them to maintain wider activity spaces. Even when facilities are limited near their homes, they may be able to reach more distant green spaces or obtain alternative recreational opportunities through travel and commercial fitness venues. They may therefore be less dependent on the local distribution of green public facilities. By contrast, socially disadvantaged groups may rely more heavily on free facilities near their homes because housing choices, time, and travel costs constrain their access to alternatives.

*Hypothesis 2*: The positive association between the spatial balance of green public facilities and residents’ health is stronger among people with lower occupational status and weakens as occupational status increases.

The potential health benefits of green public facilities are closely related to other features of urban space. Public transport can strengthen residents’ capacity to travel beyond their residential neighborhoods and access public resources. In Shanghai, Liang and Zhang (39) found that public transport accessibility to parks varied across areas in relation to park location, road networks, and transport provision. Tao et al. (40) further showed in Hong Kong that associations between public transport and activity spaces varied by income and commuting status, with bus and rail services having different implications for different groups. Residents’ daily activity spaces connect places of residence, work, care, consumption, and leisure. A larger supply of public transport stops and wider coverage across subdistricts/towns may make more distant green facilities part of residents’ potential activity spaces. Where public transport is limited, residents may depend more on nearby facilities, making uneven facility distribution more consequential for opportunities to obtain green-space resources. Public transport does not change the distribution of green facilities itself, but it may reduce residents’ dependence on their local distribution and mitigate some disadvantages that uneven provision creates for disadvantaged residents.

*Hypothesis 3*: The better the public transport connectivity, the weaker the association between the spatial balance of green public facilities and residents’ self-rated health.

## 2 Materials and methods

### 2.1 Data

Individual-level data were drawn from the 2021 Chinese General Social Survey (CGSS), conducted by the National Survey Research Center at Renmin University of China. The CGSS uses a multistage, stratified probability sampling design intended to achieve national representativeness and provides a national survey basis for studying social conditions and health inequalities in China. We linked individual survey records to district/county-level data on green public facilities, public transport, and local statistics. The final analytical sample comprised 6,871 respondents in 82 districts/counties. CGSS individual survey weights were applied in the regression analyses. Figure 1 shows the geographical distribution of the sampled districts/counties. Drawing on this national survey, the study examines associations between local resource distribution and residents’ self-rated health.

**Figure 1:**
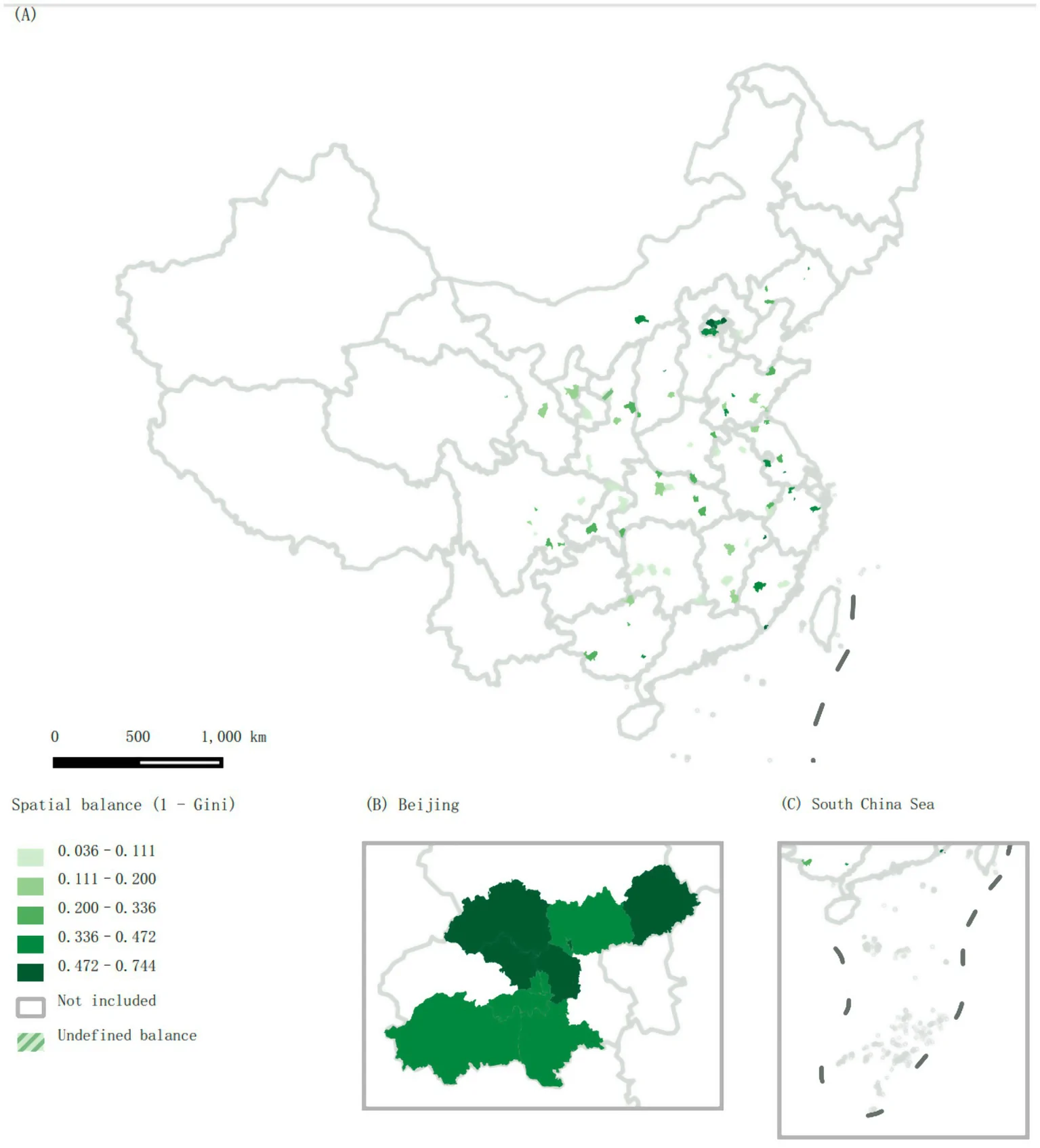
Geographical distribution of sampled districts/counties and the spatial balance of green public facilities. **(A)** National overview. **(B)** Enlarged view of Beijing. **(C)** South China Sea inset. Colors indicate spatial balance. Hatching denotes the district/county with no recorded green public facility POIs. The sample covers 82 districts/counties, of which 81 have recorded green public facility POIs. Figure 1 was created by the authors using QGIS. The administrative boundary data were obtained from DataV.GeoAtlas, which identifies Amap as its data source.

County-level administrative units were used as the contextual units of analysis. Municipal districts and counties are distinct administrative categories at the same administrative level. According to administrative division statistics at the end of 2021, China had 2,843 county-level administrative units, including 977 municipal districts, 394 county-level cities, and other categories such as counties and autonomous counties (43).

Green public facility and public transport POI records were obtained from Amap. The operational definition of green public facilities focuses on parks, community parks, small public gardens, wetland parks, forest parks, country parks, greenways, and green recreational sites intended for public use. In conceptual terms, green spaces inside schools, government institutions, residential compounds, hotels, and enterprises or public institutions fall outside this scope when they are not reliably open to the public. Similarly, a name containing terms such as green space or landscape does not, by itself, establish public access or everyday recreational use. Operational identification relied on POI names and type labels, rather than individual verification of public-access conditions. Public transport POIs mainly comprised bus stops and rail-transit stations and were used to measure district/county-level stop provision, coverage across subdistricts/towns, and rail connectivity. The identification and processing rules are reported in Supplementary Table S1, and the processing stages and record counts are reported in Supplementary Table S2.

After matching districts/counties and conducting basic data cleaning, we applied analytical inclusion criteria for self-rated health, survey weights, occupational status, and the individual- and district/county-level control variables. The final sample contained 6,871 respondents in 82 districts/counties. A total of 2,511 green public facility POI records were aggregated across 1,409 identifiable subdistricts/towns, including 862 with no identified green facilities, which were assigned a green-facility count of zero. The sampled districts/counties contained 34,256 bus-stop records and 1,379 rail-transit station records. Figure 1 shows the geographical distribution of the 82 districts/counties and their spatial balance scores. Darker shading indicates a more even distribution of green public facility POI counts across subdistricts/towns within a district/county. The sample retained 80 respondents from one district/county with no identified green public facility POIs. The original program coded its Gini coefficient as zero and its complementary spatial-balance score as one. However, when the mean facility count is zero, the denominator of the Gini formula is also zero. This coding convention cannot be interpreted as a perfectly balanced distribution. To assess sensitivity to this treatment, we re-estimated the models both with an indicator for the district/county with no recorded facilities and after excluding that district/county.

### 2.2 Variables

The dependent variable is residents’ self-rated health. It comes from the CGSS 2021 question on overall health: ‘How would you describe your current physical health?’ Responses were coded from 1 to 5, with higher values indicating better self-rated health. Self-rated health provides an overall assessment of respondents’ physical condition, functional capacity, and subjective experience of health.

The core explanatory variable is the spatial balance of green public facilities, defined as the evenness of green public facility POI counts across subdistricts/towns within each district/county. This indicator was standardized for the regression analyses, so a one-unit difference corresponds to one standard deviation in spatial balance. We aggregated green public facility POI counts within each district/county using the subdistrict/town membership fields in the original POI records. Subdistricts/towns identifiable in the data but without identified green public facility POIs were assigned a count of zero. We first calculated the spatial Gini coefficient of these counts using Equation 1, treating subdistricts/towns within each district/county as the units of calculation:

$${\text{Gini}}_{j}=\frac{1}{2n_{j}^{2}{\bar{x}}_{j}}\sum_{r=1}^{n_{j}}\sum_{s=1}^{n_{j}}|x_{\mathrm{rj}}-x_{\mathrm{sj}}|$$ (1)

where j denotes a district/county; nⱼ is the number of subdistricts/towns identified from the collected POI data and included in the calculation for district/county j; xᵣⱼ and xₛⱼ are the numbers of green public facility POIs in subdistricts/towns r and s, respectively; and x̄ⱼ is the mean number of green public facility POIs across those subdistricts/towns. The summations extend over all identified subdistricts/towns within district/county j, including those assigned zero green public facility POIs. A higher Gini coefficient indicates greater concentration of POIs in a small number of subdistricts/towns.

To align the direction of the variable with the theoretical hypothesis, the spatial Gini coefficient is reverse-coded as spatial balance in Equation 2. A higher value indicates a more balanced distribution of green public facility POIs across subdistricts/towns within a district/county:

$${\text{Balance}}_{j}=1-{\text{Gini}}_{j}$$ (2)

The models also control for green public facility supply, measured as the number of green POI records per 10,000 population, denoted by x. The regression measure was constructed by applying ln(1 + x) and then standardizing the result. Thus, the log-transformed supply value for the district/county with no recorded facilities is zero. Before standardization, the mean and sample standard deviation of the log-transformed measure were 0.257 and 0.188, respectively. These standardization parameters were fixed before selection of the final analytical sample.

Two main moderating variables are included: occupational status and public transport connectivity. Occupational status is measured using the International Socio-Economic Index of Occupational Status (ISEI), with higher values indicating higher occupational status.

Public transport connectivity combines bus-stop and rail-transit station densities with their coverage across subdistricts/towns. Density is measured as the number of station or stop records per square kilometer of administrative area, with ln(1 + density) applied separately to bus and rail densities. The two log-density measures and two coverage measures were each standardized across the 84 environmental units in the source data using the population standard deviation. The basic bus-connectivity index is the mean of the two standardized bus measures, and the composite bus-and-rail index is the equally weighted mean of all four standardized measures. The composite index was standardized again in the matched data before entering the regression models. Bus-stop density, bus-stop coverage, the basic bus-connectivity index, and the standardized composite index are defined in Equations 3–6, respectively:

$${\text{BusDensity}}_{j}=\frac{{\text{BusPOI}}_{j}}{{\text{Area}}_{j}}$$ (3)

$${\text{BusCoverage}}_{j}=\frac{{\text{BusTown}}_{j}}{{\text{Town}}_{j}}$$ (4)

$${\text{PTBus}}_{j}=\frac{Z[\ln(1+{\text{BusDensity}}_{j})]+Z({\text{BusCoverage}}_{j})}{2}$$ (5)

$${\mathrm{PT}}_{j}=Z({\text{PTMetroIndex}}_{j})$$ (6)

where BusPOIⱼ is the number of bus-stop records in district/county j; Areaⱼ is its administrative area in square kilometers; BusTownⱼ is the number of its subdistricts/towns with at least one bus stop; and Townⱼ is the number of identifiable subdistricts/towns. In Equation 5, Z(·) denotes standardization across the 84 source environmental units using the population standard deviation, and PTBusⱼ is the basic bus-connectivity index. PTMetroIndexⱼ is the equally weighted mean of standardized log bus-stop density, standardized bus-stop coverage, standardized log rail-transit station density, and standardized rail-transit coverage. In Equation 6, Z(·) denotes the further standardization of this composite index in the matched data, yielding PTⱼ. Higher values indicate greater district/county-level stop and station provision per square kilometer and broader coverage across subdistricts/towns.

Individual-level controls include age, sex, years of education, household registration status, marital status, Communist Party of China membership, and ethnicity. District/county-level controls include log-transformed GDP per capita and the log-transformed number of medical beds per 10,000 population. The medical-bed measure retains imputations already present in the source data. Values for 29 districts/counties were imputed using the median among districts/counties with observed values in the same province; where no observed values were available within a province, the median across all 55 source units with observed values was used. The two main sets of models additionally control for the natural logarithm of the resident population and the natural logarithm of administrative area, respectively. Population is measured in persons and area in square kilometers. Models adjusting simultaneously for population and area are reported in Supplementary Table S3.

Figure 2 presents the joint distribution of green public facility supply and spatial balance. Low-supply/low-balance and high-supply/high-balance districts/counties are the main types in the sample, but high-supply/low-balance and low-supply/high-balance cases also occur. The two measures therefore do not correspond uniformly. A district/county with more green public facilities does not necessarily distribute them evenly across subdistricts/towns. Conversely, a district/county with relatively limited provision may exhibit higher spatial balance because its facilities are more dispersed. The district/county with no recorded facilities represents a case in which the available POI data identified no green facilities meeting the study criteria, and cannot be interpreted as having an ideally balanced distribution.

**Figure 2:**
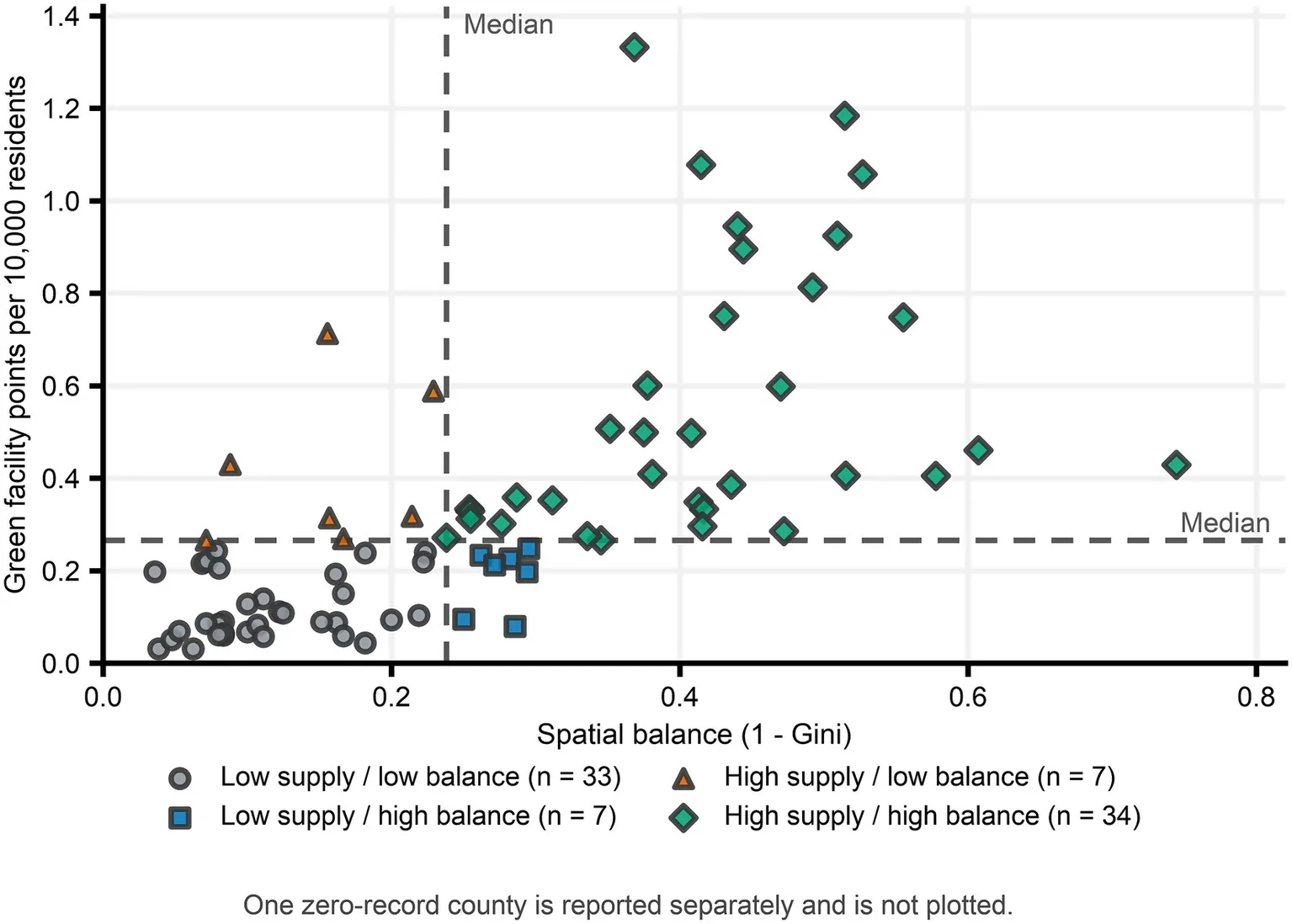
District/county types by green public facility supply and spatial balance. Each point represents one of the 81 districts/counties with recorded green public facility POIs. The dividing lines are the medians calculated with equal weight for each district/county: 0.238 for spatial balance and 0.266 for green public facility POIs per 10,000 population.

### 2.3 Analytical models

The analysis examines conditional associations between the distribution of green public facilities and individual self-rated health, and whether these associations vary with occupational status and public transport connectivity. We used ordinary least squares (OLS) regression with CGSS individual survey weights to express these associations and interactions on the original 1–5 health-score scale. Multilevel modelling is also appropriate for hierarchical data. We retained weighted OLS as the main approach because our primary interest was in these regression associations, rather than district/county-specific predictions or variance decomposition. To account for dependence among respondents within districts/counties, all main models use district/county-clustered robust standard errors. This approach allows within-district/county error correlation and heteroskedasticity without specifying a particular within-cluster correlation structure. The choice therefore reflects the analytical objective and approach to statistical inference. The core model is specified in Equation 7:

$$\begin{array}{l} {\text{Health}}_{\mathrm{ij}}=\beta_{0}+\beta_{1}{\text{Balance}}_{j}+\beta_{2}{\text{Supply}}_{j}+\beta_{3}{\mathrm{PT}}_{j}\\ +\beta_{4}{\text{ISEI}}_{\mathrm{ij}}+\beta_{5}({\text{Balance}}_{j}\times {\text{ISEI}}_{\mathrm{ij}})\\ +\beta_{6}({\text{Balance}}_{j}\times {\mathrm{PT}}_{j})+\gamma X_{\mathrm{ij}}+\delta Z_{j}+\varepsilon_{\mathrm{ij}} \end{array}$$ (7)

where Health_ij_ is the self-rated health of individual i in district/county j; Balance_j_ is the spatial balance of green public facilities in district/county j; Supply_j_ is the level of green public facility provision in district/county j; PT_j_ is public transport connectivity in district/county j; ISEI_ij_ is the occupational status of individual i; X_ij_ is a vector of individual-level control variables; Z_j_ is a vector of district/county-level control variables; and ε_ij_ is the error term. β_0_ is the intercept; β_1_–β_6_ are coefficients for the focal variables and interaction terms; and γ and δ are coefficient vectors. The focal continuous predictors, including Supply, Balance, PT, and ISEI, are standardized, while self-rated health remains on its original 1–5 scale. The interaction coefficients therefore indicate how the association between spatial balance and health changes with occupational status or public transport connectivity.

Because self-rated health is an ordered categorical variable, ordered logit models were estimated as robustness checks. The stability of the main findings was also examined using unweighted OLS models, alternative spatial indicators, and alternative treatments of the district/county with no recorded facilities. A null model yielded a district/county-level intraclass correlation coefficient (ICC) of approximately 0.047, indicating some correlation in health ratings among respondents within the same district/county. Estimates for district/county-level variables primarily depend on variation across the 82 districts/counties, so the statistical information available for these estimates should not be equated with 6,871 independent individual observations. We therefore consider coefficient magnitudes and confidence intervals and interpret results close to conventional significance thresholds cautiously.

## 3 Results

### 3.1 Descriptive statistics

Table 1 reports descriptive statistics for the main variables. The analytical sample contains 6,871 respondents in 82 districts/counties. Mean self-rated health is 3.433, with a standard deviation of 1.091, indicating that respondents’ overall health assessments are moderately high but vary substantially among individuals. The mean number of green public facility POIs per 10,000 population is 0.315. Mean spatial balance is 0.260, indicating that green public facility POIs remain concentrated in a small number of subdistricts/towns in many sampled districts/counties.

**Table 1:** Descriptive statistics for the main variables.

Variable	*N*	Mean	SD	Minimum	Maximum
Self-rated health	6,871	3.433	1.091	1.000	5.000
Green public facility POIs per 10,000 population	6,871	0.315	0.277	0.000	1.332
Spatial balance	6,871	0.260	0.178	0.036	1.000
Public transport connectivity	6,871	−0.007	0.867	−0.941	3.347
Occupational status (ISEI)	6,871	32.162	17.498	10.000	89.000
Age	6,871	53.520	16.335	18.000	99.000
Sex	6,871	0.456	0.498	0.000	1.000
Years of education	6,871	9.060	4.554	0.000	19.000
Household registration status	6,871	0.395	0.489	0.000	1.000
Marital status	6,871	0.753	0.431	0.000	1.000
Communist Party of China membership	6,871	0.124	0.329	0.000	1.000
Ethnicity	6,871	0.927	0.260	0.000	1.000
Log GDP per capita	6,871	10.690	2.006	3.434	13.100
Log medical beds per 10,000 population	6,871	4.154	0.420	3.122	5.171
Log resident population	6,871	13.180	0.702	11.158	15.051
Log administrative area	6,871	6.917	1.094	3.738	8.642

Spatial balance equals 1 minus the spatial Gini coefficient of green public facilities. Sex, household registration status, marital status, Communist Party of China membership, and ethnicity are coded as binary variables from 0 to 1.

### 3.2 Regression results

Table 2 reports OLS estimates of the relationship between the spatial balance of green public facilities and residents’ self-rated health. Model 1 examines the basic association between spatial balance and self-rated health. Model 2 adds the interaction between spatial balance and public transport connectivity. Model 3 adds the interaction between spatial balance and occupational status measured by ISEI. Model 4 includes both interactions. All four models control for green public facility supply, individual sociodemographic characteristics, and district/county development conditions. District/county-clustered robust standard errors are reported in parentheses. Table 2 contains separate panels adjusting for population and administrative area, respectively. Both panels use the log-transformed and standardized measure of green public facility supply.

**Table 2:** OLS regression models of residents’ self-rated health.

A. Models adjusted for population size				
Variable	Model 1	Model 2	Model 3	Model 4
Green facility supply (log-transformed, standardized)	−0.040 (0.032)	−0.033 (0.031)	−0.044 (0.031)	−0.038 (0.031)
Spatial balance	0.059* (0.026)	0.034 (0.023)	0.071* (0.027)	0.048* (0.024)
Public transport connectivity	−0.013 (0.032)	0.027 (0.036)	−0.008 (0.032)	0.026 (0.036)
Occupational status (ISEI)	0.086*** (0.019)	0.086*** (0.019)	0.078*** (0.019)	0.079*** (0.019)
Spatial balance × public transport	—	−0.059** (0.022)	—	−0.051* (0.023)
Spatial balance × ISEI	—	—	−0.057** (0.020)	−0.052** (0.019)
Log resident population	0.028 (0.038)	0.037 (0.038)	0.030 (0.038)	0.037 (0.038)
Individual-level controls	Yes	Yes	Yes	Yes
District/county-level controls	Yes	Yes	Yes	Yes
*N*	6,871	6,871	6,871	6,871
Districts/counties	82	82	82	82
*R* ^2^	0.141	0.143	0.143	0.144

B. Models adjusted for administrative area				
Variable	Model 1	Model 2	Model 3	Model 4
Green facility supply (log-transformed, standardized)	−0.051 (0.033)	−0.047 (0.031)	−0.055^+^ (0.032)	−0.051^+^ (0.030)
Spatial balance	0.051^+^ (0.028)	0.024 (0.024)	0.062* (0.029)	0.037 (0.026)
Public transport connectivity	−0.070 (0.047)	−0.028 (0.047)	−0.062 (0.047)	−0.026 (0.047)
Occupational status (ISEI)	0.084*** (0.019)	0.084*** (0.019)	0.077*** (0.019)	0.078*** (0.019)
Spatial balance × public transport	—	−0.063** (0.022)	—	−0.056* (0.023)
Spatial balance × ISEI	—	—	−0.053** (0.019)	−0.048* (0.018)
Log administrative area	−0.094* (0.046)	−0.101* (0.047)	−0.089^+^ (0.045)	−0.096* (0.046)
Individual-level controls	Yes	Yes	Yes	Yes
Other district/county-level controls	Yes	Yes	Yes	Yes
*N*	6,871	6,871	6,871	6,871
Districts/counties	82	82	82	82
*R* ^2^	0.143	0.145	0.145	0.147

District/county-clustered robust standard errors are in parentheses. ^+^*p* < 0.10, *p < 0.05, **p < 0.01, ***p < 0.001. All models use CGSS individual survey weights and control for age, sex, household registration status, marital status, Communist Party of China membership, ethnicity, years of education, log GDP per capita, and log medical beds per 10,000 population. Panel A additionally adjusts for the natural logarithm of the resident population, and Panel B for the natural logarithm of administrative area. Supply is log-transformed and standardized; spatial balance, public transport connectivity, and ISEI are also standardized. In interaction models, the spatial-balance main term represents the conditional association when the moderator or moderators included in the interactions equal zero on their standardized scales.

The coefficients for green public facility supply are negative in both sets of models, ranging from −0.044 to −0.033 in the population-adjusted models and from −0.055 to −0.047 in the area-adjusted models. None reaches the 5% significance level. The supply coefficients in Models 3 and 4 of the area-adjusted panel reach only the 10% level. Thus, given the study’s measures, analytical sample, and covariate adjustments, the results do not provide stable evidence of an association between facility supply and self-rated health. Spatial balance, by contrast, is positively associated with self-rated health in some specifications. Facility counts and their spatial distribution may therefore capture different environmental characteristics, supporting consideration of both supply and its distribution within districts/counties.

In the population-adjusted Model 1, the coefficient for spatial balance is 0.059 and is significant at the 5% level. A one-standard-deviation higher spatial-balance score is thus associated with a 0.059-point higher predicted self-rated health score on the 1–5 scale. This is a small difference in absolute terms. In the area-adjusted Model 1, the coefficient is 0.051 and reaches only the 10% significance level. These estimates provide conditional support for Hypothesis 1, with the strength of evidence depending on the model specification.

#### 3.2.1 Moderating role of occupational status

In the population-adjusted models, the spatial balance × ISEI interaction coefficients are −0.057 in Model 3 and −0.052 in Model 4, both significant at the 1% level. The corresponding coefficients in the area-adjusted models are −0.053 and −0.048, significant at the 1 and 5% levels, respectively. Holding other variables constant in Model 4, a one-standard-deviation higher ISEI score reduces the predicted self-rated health difference associated with a one-standard-deviation increase in spatial balance by 0.052 points in the population-adjusted model and 0.048 points in the area-adjusted model. The negative interactions indicate that the positive association between spatial balance and self-rated health weakens as occupational status increases. Figure 3 presents the marginal association of spatial balance with self-rated health at different ISEI values, calculated from Model 4 in both panels of Table 2. The horizontal axis shows standardized ISEI, and the vertical axis shows the predicted change in self-rated health associated with a one-standard-deviation increase in spatial balance. Shaded areas denote 95% confidence intervals.

**Figure 3:**
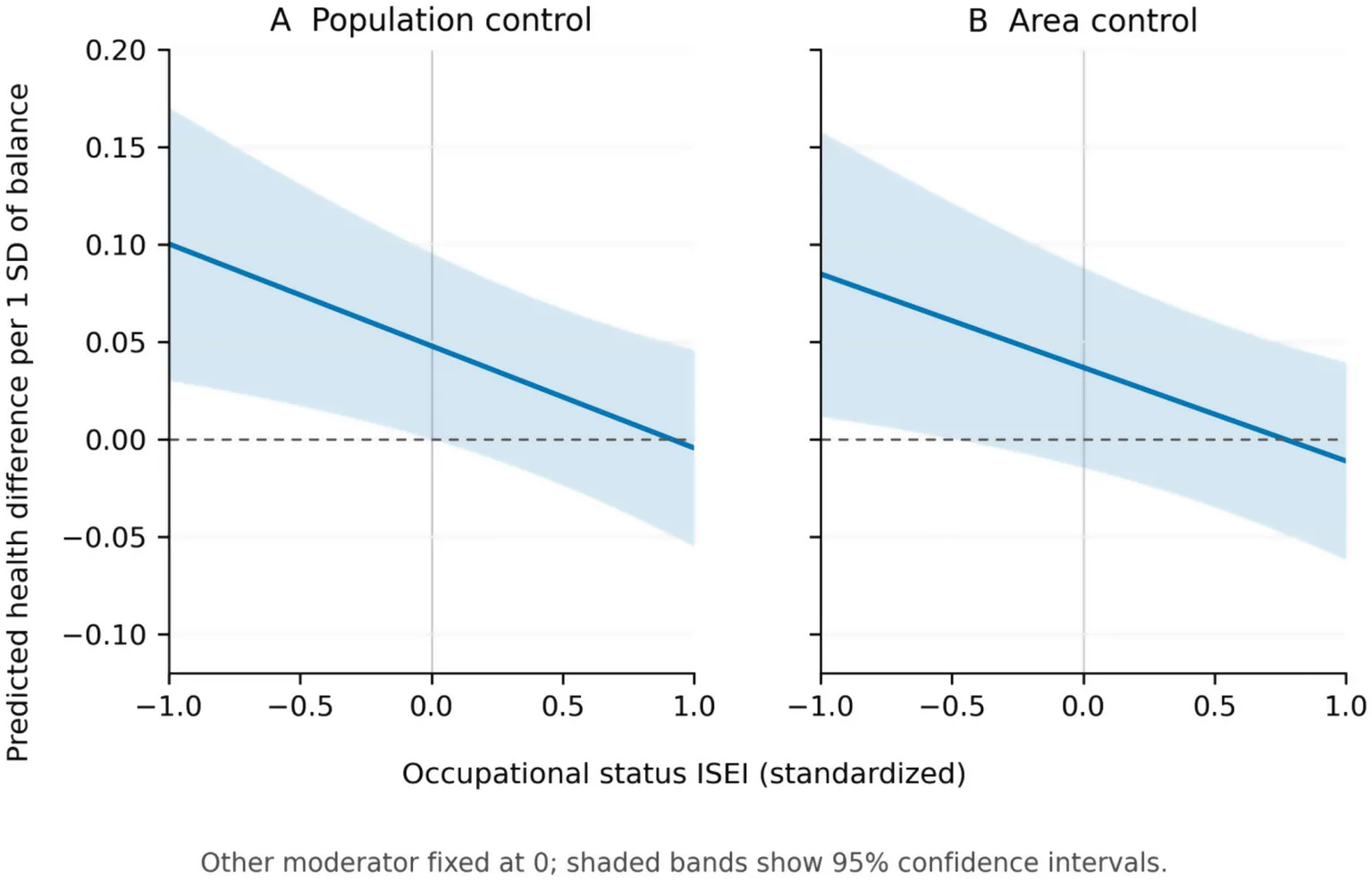
Marginal associations of spatial balance with self-rated health by occupational status. Panel **(A)** shows estimates from Model 4 adjusted for population size, and panel **(B)** shows estimates from Model 4 adjusted for administrative area (Table 2). The horizontal axis shows standardized occupational status (ISEI). The vertical axis shows the marginal association of spatial balance with self-rated health. Shaded areas denote 95% confidence intervals calculated using district/county-clustered robust standard errors; the dashed line marks zero. A standardized value of zero denotes the reference point used for standardization.

Figure 3 shows that the positive marginal association between spatial balance and self-rated health is stronger at lower levels of occupational status and gradually weakens as occupational status rises, approaching zero at higher levels. This pattern suggests that the spatial distribution of green public facilities is more strongly associated with health among residents with lower occupational status. One possible interpretation is that constraints on mobility, residential choice, and access to leisure resources make these residents more dependent on nearby facilities accessible at low cost. A more even distribution across subdistricts/towns could then provide greater opportunities for everyday exercise, leisure, psychological restoration, and social interaction. These mechanisms were not directly tested, but the observed interaction is consistent with Hypothesis 2 and points to the potential relevance of facility distribution for health equity.

#### 3.2.2 Moderating role of public transport connectivity

The spatial balance × public transport interaction is negative in both sets of models, reaching the 1% significance level in Model 2 and the 5% level in Model 4. The Model 4 interaction coefficients are −0.051 in the population-adjusted model and −0.056 in the area-adjusted model. Holding other variables constant, a one-standard-deviation higher public transport connectivity score reduces the predicted self-rated health difference associated with a one-standard-deviation increase in spatial balance by 0.051 and 0.056 points, respectively. These estimates indicate that the association between spatial balance and self-rated health becomes weaker at higher levels of public transport connectivity. Figure 4 plots the marginal association of spatial balance with self-rated health at different levels of public transport connectivity, based on Model 4 in both panels.

**Figure 4:**
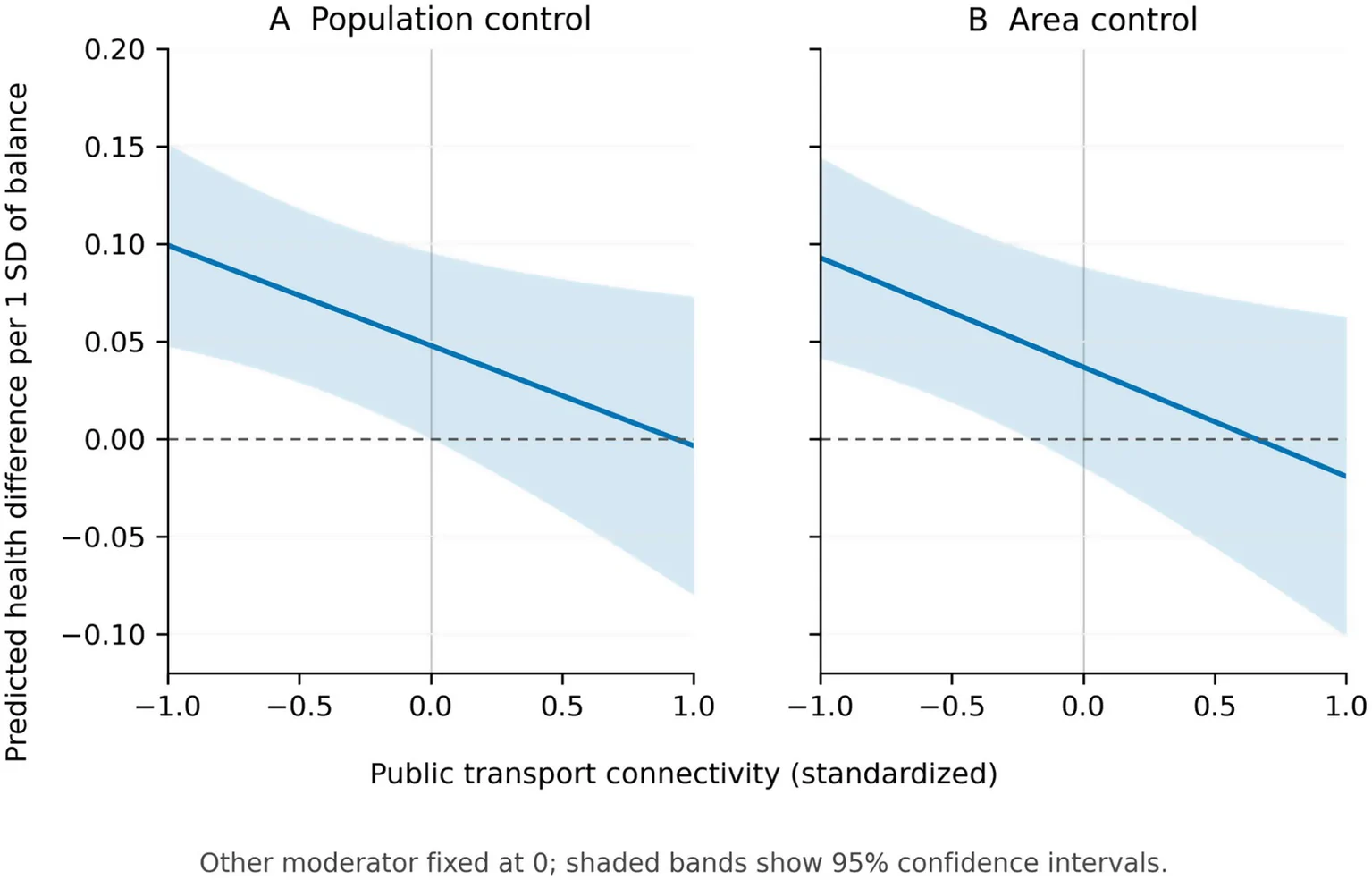
Marginal associations of spatial balance with self-rated health by public transport connectivity. Panel **(A)** shows estimates from Model 4 adjusted for population size, and panel **(B)** shows estimates from Model 4 adjusted for administrative area (Table 2). The horizontal axis shows standardized public transport connectivity. The vertical axis shows the marginal association of spatial balance with self-rated health. Shaded areas denote 95% confidence intervals calculated using district/county-clustered robust standard errors; the dashed line marks zero. A standardized value of zero denotes the reference point used for standardization.

Figure 4 shows a positive, statistically significant marginal association between spatial balance and self-rated health at lower levels of public transport connectivity. This association gradually weakens as connectivity increases. One possible explanation is that better public transport expands residents’ daily activity spaces and reduces their dependence on the distribution of facilities within their own district/county. The interaction does not establish that public transport lowers health or directly identify its effect on health. Instead, it indicates that the association between facility distribution and health varies with transport conditions. The main models support Hypothesis 3, but the robustness checks below show that this evidence is sensitive to the choice of indicators and the treatment of the district/county with no recorded facilities.

### 3.3 Robustness checks

To assess whether the findings depend on particular model specifications or variable treatments, several supplementary models were estimated as robustness checks.

First, unweighted OLS and ordered logit models were estimated in place of the main model. Second, the basic bus-connectivity index replaced the composite bus-and-rail connectivity measure. As specified in Equation 5, the basic index is the mean of standardized log bus-stop density per square kilometer and standardized bus-stop coverage across subdistricts/towns; it was then standardized again in the matched data. Third, the models were re-estimated using two alternative spatial indicators. The first is based on the coefficient of variation of green public facility POI counts across subdistricts/towns within each district/county and is reverse-coded so that higher values represent a more even distribution. The second measures coverage as the proportion of identifiable subdistricts/towns within a district/county with at least one green public facility POI. Higher values indicate broader coverage. Fourth, the models were re-estimated both with a dummy variable for the district/county with no recorded facilities and after excluding that district/county. These checks compare the direction, magnitude, and statistical uncertainty of the occupational-status and public-transport interactions across model specifications and indicators.

As shown in Table 3, unweighted OLS and ordered logit estimates of spatial balance and both interactions retain the directions observed in the main models. The spatial-balance main term reaches the 5% significance level in the population-adjusted models but only the 10% level in the area-adjusted models. Both interactions remain significant at the 5% level in these checks. Replacing the composite bus-and-rail measure with the basic bus-connectivity index leaves the public transport interaction negative, but it reaches only the 10% significance level in the population-adjusted model; it remains significant at the 5% level in the area-adjusted model. The occupational-status interaction remains significant in both panels.

**Table 3:** Robustness checks and treatment of the district/county with no recorded facilities.

A. Models adjusted for population size				
Model or treatment	Spatial indicator	Spatial indicator × public transport	Spatial indicator × ISEI	*N*
Main model	0.048* (0.024)	−0.051* (0.023)	−0.052** (0.019)	6,871
Unweighted OLS	0.052* (0.021)	−0.063** (0.019)	−0.048** (0.016)	6,871
Ordered logit	0.112* (0.045)	−0.095* (0.040)	−0.091* (0.036)	6,871
Basic bus-connectivity index	0.040 (0.027)	−0.035^+^ (0.020)	−0.054** (0.019)	6,871
Reverse-coded coefficient-of-variation indicator	0.005 (0.044)	−0.050 (0.048)	−0.038* (0.019)	6,871
Township/subdistrict coverage indicator	0.032 (0.038)	−0.036 (0.030)	−0.064** (0.020)	6,871
Zero-record indicator included	0.033 (0.035)	−0.042 (0.026)	−0.055** (0.019)	6,871
Zero-record districts/counties excluded	0.033 (0.035)	−0.040 (0.027)	−0.061** (0.023)	6,791

B. Models adjusted for administrative area				
Model or treatment	Spatial indicator	Spatial indicator × public transport	Spatial indicator × ISEI	*N*
Main model	0.037 (0.026)	−0.056* (0.023)	−0.048* (0.018)	6,871
Unweighted OLS	0.040^+^ (0.022)	−0.069*** (0.018)	−0.044** (0.016)	6,871
Ordered logit	0.091^+^ (0.049)	−0.103* (0.040)	−0.084* (0.035)	6,871
Basic bus-connectivity index	0.030 (0.028)	−0.045* (0.020)	−0.052** (0.018)	6,871
Reverse-coded coefficient-of-variation indicator	−0.017 (0.045)	−0.067 (0.049)	−0.036* (0.018)	6,871
Township/subdistrict coverage indicator	0.029 (0.040)	−0.038 (0.030)	−0.061** (0.019)	6,871
Zero-record indicator included	0.021 (0.036)	−0.045^+^ (0.027)	−0.051** (0.018)	6,871
Zero-record districts/counties excluded	0.022 (0.036)	−0.044 (0.027)	−0.056* (0.021)	6,791

District/county-clustered robust standard errors are in parentheses. All models include both interactions and use the log-transformed supply measure and other controls specified in Table 2. Panels A and B adjust for population and area, respectively. All models except unweighted OLS use CGSS individual survey weights. Alternative spatial indicators are oriented so that higher values indicate a more even distribution or broader coverage. The spatial-indicator main term is a conditional association in an interaction model. Ordered logit and OLS coefficients are expressed on different scales. Excluding the district/county with no recorded facilities leaves 6,791 respondents in 81 districts/counties; the remaining models use 6,871 respondents in 82 districts/counties. ^+^*p* < 0.10, *p < 0.05, **p < 0.01, ***p < 0.001.

Across all robustness checks, the occupational-status interaction remains negative and significant at the 5% level. The public transport interaction is also consistently negative. It remains significant in the unweighted OLS and ordered logit models; with the basic bus-connectivity index, it reaches only the 10% level in the population-adjusted model but remains significant at the 5% level in the area-adjusted model. When the spatial measure is replaced by the reverse-coded coefficient of variation or subdistrict/town coverage, and when an indicator for the district/county with no recorded facilities is added or that district/county is excluded, the public transport interaction does not reach the 5% significance level. The spatial-balance main term is similarly sensitive to alternative indicators and the treatment of the district/county with no recorded facilities. Hypothesis 2 therefore receives relatively stable support, whereas support for Hypotheses 1 and 3 is conditional. The direction of the public transport interaction is consistent, but its estimated magnitude is subject to greater statistical uncertainty.

Figure 5 compares estimates of the two interactions across specifications. Circles indicate the interaction between spatial balance and public transport connectivity, squares indicate the interaction between spatial balance and ISEI, and horizontal lines show 95% confidence intervals. The occupational-status interaction is negative throughout, and none of its 95% confidence intervals crosses zero. The public transport interaction is also consistently negative. With alternative spatial indicators or either adjustment for the district/county with no recorded facilities, its 95% confidence intervals cross zero in both panels. With the basic bus-connectivity index, the confidence interval crosses zero in the population-adjusted panel but not in the area-adjusted panel. Because ordered logit and OLS coefficients are on different scales, the figure includes only OLS estimates; ordered logit results are reported in Table 3. The figure illustrates the direction and statistical uncertainty of the estimates across the robustness checks.

**Figure 5:**
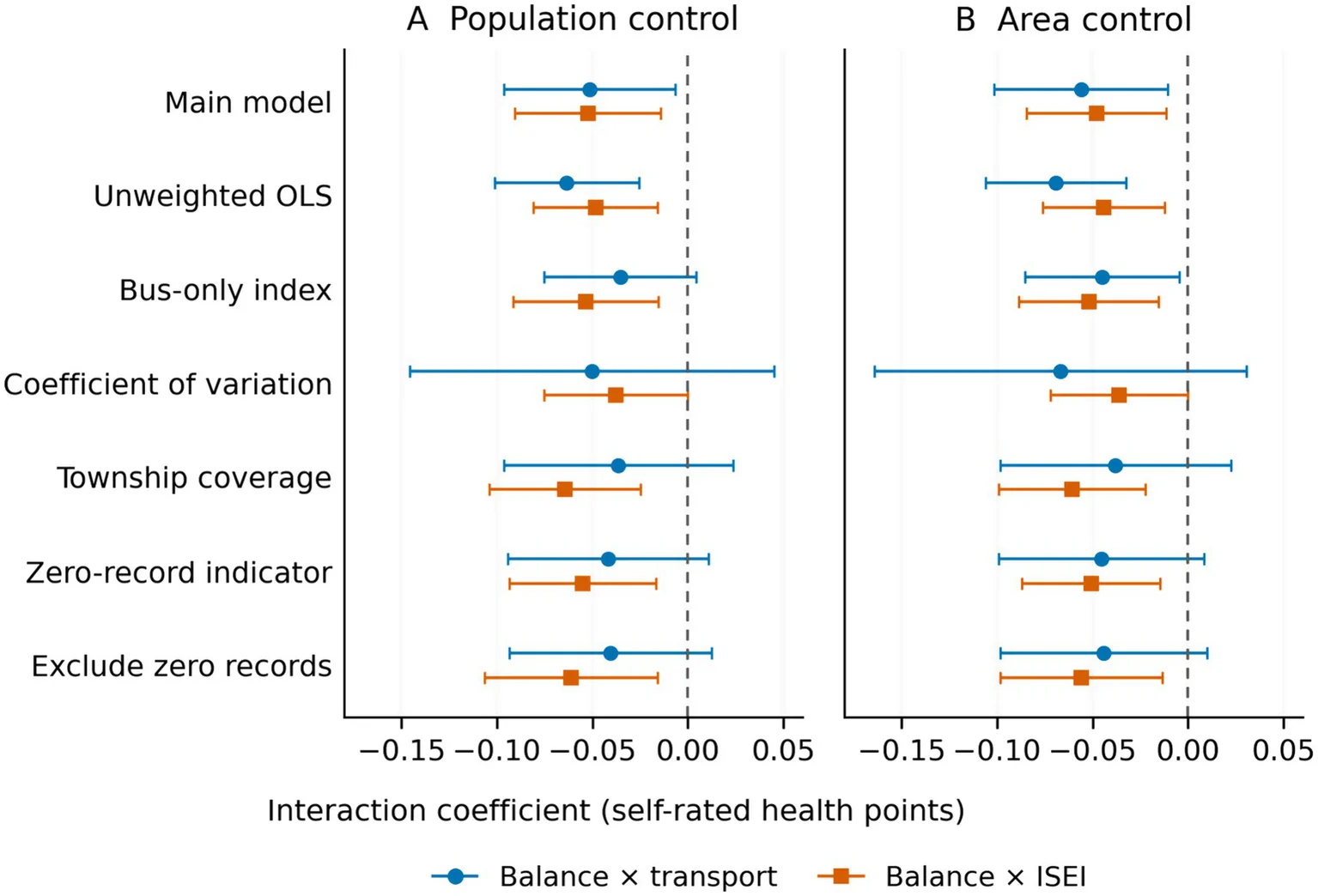
Interaction coefficients and 95% confidence intervals across model specifications. Panels **(A,B)** adjust for population and area, respectively, and correspond to the OLS models in Table 3. Circles indicate the spatial balance × public transport interaction; squares indicate the spatial balance × ISEI interaction. Horizontal lines denote 95% confidence intervals, and the dashed line marks zero. Ordered logit results are reported only in Table 3.

## 4 Discussion

From a socio-spatial perspective, this study conceptualizes green public facilities as green-space resources embedded in the urban public-resource system. It examines the relationship between the spatial distribution of green public facilities within cities and residents’ health and further analyzes how this relationship varies with occupational status and public transport conditions. The study emphasizes that the potential health benefits of green public facilities need to be understood in relation to spatial distribution, the production of space, and spatial accessibility, rather than assumed to follow automatically from facility provision.

First, the findings suggest that spatial distribution merits consideration alongside the amount of green public facilities provided. Jarvis et al. (22) found only a weak correlation between public green-space accessibility and natural land cover around residences. The POI-based indicators used here also differ from vegetation cover, facility area, and actual use. The coefficients for facility supply are negative in all main models, but none is statistically significant at the 5% level. The analysis therefore provides no evidence of a stable association between facility supply and residents’ self-rated health. After adjusting for supply, spatial balance is positively associated with self-rated health in only some models, and the association is small and sensitive to model specification. These findings suggest that studies of green public facilities and health should consider how facilities are distributed across subdistricts/towns within districts/counties in addition to the scale of provision. Total provision and spatial distribution capture different dimensions of public-resource allocation.

Second, the association between the spatial distribution of green public facilities and residents’ health differs by occupational status. This is the study’s most robust empirical finding. The positive association between spatial balance and self-rated health is more pronounced among respondents with lower occupational status. One possible explanation is that people with higher occupational status have greater environmental bargaining power, discretionary time, mobility, or access to alternative resources, making them less dependent on the distribution of green public facilities within their district/county. People with lower occupational status may rely more on nearby public facilities that can be reached at low cost. A more balanced distribution across subdistricts/towns could therefore offer them more opportunities for everyday exercise, leisure, psychological restoration, and social interaction. These explanations were not directly tested in this study. Nevertheless, the observed differences suggest that the spatial distribution of green public facilities is relevant not only to urban planning but also to the distribution of health opportunities across social groups.

Third, public transport conditions may moderate the relationship between the spatial balance of green public facilities and residents’ health. In the main models, the positive association between spatial balance and self-rated health weakens as public transport connectivity improves. Public transport may expand residents’ everyday activity spaces and reduce their dependence on a balanced distribution of facilities within their own district/county. However, this finding is sensitive to the choice of indicators and the treatment of districts/counties with no recorded green public facility POIs. It is less robust than the occupational-status interaction. Future research should distinguish route accessibility, travel-time accessibility, transport connections around facilities, and residents’ actual travel behavior.

Taken together, the findings support examining green public facilities as public health resources situated within social space. The observed variation by occupational status is consistent with the theoretical argument that potential health benefits depend on the social and spatial conditions under which resources are available and used. Uneven facility distribution may intersect with social disadvantage, although this study does not establish that spatial inequality causes or reproduces social inequality. The provision and formal availability of public resources may not ensure practical access, and access alone may not ensure health benefits. Green public facilities thus provide a useful setting for examining how health opportunities are distributed in social space and for connecting urban planning and public health research with sociological debates on the production of space, social stratification, and health inequality.

The study also has policy implications. Given the small observed associations and the inability of this cross-sectional analysis to identify causal relationships, the following suggestions are directions for further planning assessment. Specific measures require consideration of local needs, implementation costs, and subsequent evaluation. Alongside the supply of green public facilities, healthy-city initiatives could assess their distribution across subdistricts/towns within districts/counties and the conditions that enable disadvantaged groups to benefit from these resources. Assessments could combine population needs, facility area and quality, and travel conditions to identify areas where social disadvantage, insufficient public green resources, and weak transport connectivity may overlap. They could then examine the feasibility of improving affordable everyday access in these areas. Community parks, pocket parks, roadside green spaces, connected greenways, walking and cycling networks, and links to public transport are possible options for such assessments.

### 4.1 Limitations

First, the cross-sectional observational data cannot establish the temporal ordering of facility distribution and health or rule out reverse causation, residential self-selection, and unobserved confounding. The results should therefore be interpreted as statistical associations. Self-rated health may also be affected by differences in personal evaluation standards and reporting. Second, spatial balance captures the distribution of green public facility POI counts across subdistricts/towns. It does not incorporate facility area, quality, service capacity, maintenance, or opening conditions, and it is not weighted by subdistrict/town population. A balanced distribution of counts therefore does not necessarily imply balanced service capacity or equitable access.

Population and geographic heterogeneity across districts/counties may affect the interpretation and comparability of this measure. Facilities concentrated in more populous subdistricts/towns may yield a lower balance score even when their distribution broadly follows the population distribution. Conversely, similar balance scores may correspond to different travel distances and service coverage in districts/counties of different geographic sizes. Differences in the number and boundaries of subdistricts/towns may also affect the measured distribution of facility counts. Adjusting for district/county population and administrative area in the regression models controls for overall differences in these characteristics but does not make the balance measure population- or area-adjusted. Subdistricts/towns are identified from the available POI records, and their coverage may also affect measurement. Third, respondents in the same district/county are assigned the same facility-allocation indicators, although their distance from facilities, activity spaces, and actual use may differ. The study examines district/county-level resource-allocation contexts in relation to individual health and cannot identify respondents’ actual green-space exposure. Although the CGSS uses a nationally representative sampling design, spatial matching and the treatment of missing variables may have changed the composition of the final analytical sample. Generalization to areas outside the sample therefore requires caution.

## Data Availability

The survey data analysed in this study are from the publicly released 2021 Chinese General Social Survey (CGSS). Researchers should apply directly to the data provider through the official CGSS data-access procedures (https://cgss.ruc.edu.cn/) and comply with the applicable registration, access and use conditions. The linked individual-level analysis data combine CGSS respondent records with district/county-level contextual data. These linked data are not available for unrestricted redistribution because access to the underlying CGSS microdata and disclosure of restricted geographic information are governed by the data provider’s permissions and confidentiality requirements. Any sharing of linked data must comply with those requirements and any required third-party permissions. Requests concerning the linked analysis data or other data underlying this study should be directed to the corresponding author at wangyuanchao@sdu.edu.cn and are subject to the applicable permissions and conditions of the original data providers.
