# Supplementary material for: Green public facilities and residents’ health from a socio-spatial perspective
Source: Front Public Health. 2026 Oct 30;14:1972132. doi: 10.3389/fpubh.2026.1972132 (PMC13759749; doi:10.3389/fpubh.2026.1972132)
Supplement: Supplementary file 1 [file Data_Sheet_1.PDF]

Supplementary Table S1. Rules for identifying and processing green facility POIs

Processing stage	Rule
Exact duplicate records	Within each source file, remove records with identical values for name, x, y, type, adcode, 镇街名 (subdistrict/town name), and 村居名 (village/community name), using the fields present in the file. Retain the first record.
Type criterion	Match the type category 风景名胜;公园广场 (scenic spots; parks and squares), including 公园 (park), 植物园 (botanical garden), or 公园内部设施 (facilities within parks).
Name criterion	Match names containing any of these Chinese strings: 公园 (park), 游园 (small public garden), 植物园 (botanical garden), 湿地公园 (wetland park), 湿地 (wetland), 森林公园 (forest park), 郊野公园 (country park), 绿地 (green space), 风景区 (scenic area), 风景名胜 (scenic spot), 旅游区 (tourist area), 景区 (scenic attraction), 自然保护 (nature conservation), or 水利风景 (water-conservancy landscape).
Combined type and name criterion	Match types containing 风景名胜 (scenic spots) or 公园景点售票处 (park/attraction ticket office), together with names containing any of these strings: 公园 (park), 游园 (small public garden), 植物园 (botanical garden), 湿地 (wetland), 森林 (forest), 郊野 (countryside), 风景区 (scenic area), 景区 (scenic attraction), 水利风景 (water-conservancy landscape), 山 (mountain), 湖 (lake), 河 (river), 江 (large river), 海 (sea), 峡 (gorge), or 漂流 (rafting).
Combination of rules	Assign a green-facility indicator of 1 when any identification criterion is met. A record meeting several criteria is counted only once.
Subdistrict/town aggregation	Sum green-facility indicators by the existing subdistrict/town field within each district/county file. Include all subdistricts/towns identifiable in the collected POI records. Assign zero green facilities to an identified subdistrict/town when its indicator sum is zero.
Distribution measure	Calculate the Gini coefficient of subdistrict/town green-facility POI counts without weighting by population or facility area. Spatial balance equals 1 minus this Gini coefficient.
Zero-record coding	The original program assigns a Gini coefficient of 0 to a district/county with no green-facility records, yielding a spatial-balance value of 1. The Gini formula is undefined in this case; this coding convention does not represent ideal balance.
Unit and scope of measurement	The unit is an identified POI record, which does not necessarily correspond to a distinct park. Exact-record deduplication does not merge entrances, internal facilities, or nearby records belonging to the same place. Name and type matching does not verify public access at each location.

Note: Chinese strings are retained to specify the matching rules; parenthetical English text provides glosses. A name matches when it contains a listed string. The measure describes identified POI-count distributions, rather than facility area, quality, use, or complete administrative coverage.

Supplementary Table S2. POI processing and analytical sample selection

Stage	Description	Count
**A. POI processing in the 82 districts/counties in the analytical sample**		
Source records	Original POI records	37,672
Exact duplicates	Records removed	11,055
After deduplication	Records retained	26,617
Green-facility identification	Records meeting the rules in Table S1	2,511
Subdistricts/towns	Identifiable subdistricts/towns included in aggregation	1,409
Zero green-facility counts	Identified subdistricts/towns with no green-facility records	862
Districts/counties	All analytical units / units with green-facility records	82 / 81
Bus stops	Records included in transport indicators	34,256
Metro stations	Records included in the composite transport indicator	1,379
Transport hubs	Records excluded from both transport indicators	255
**B. Sequential screening of individual records in the matched model file**		
Starting records	Individual records before analytical screening	8,038
Self-rated health	Excluded at this stage	6
Years of education	Excluded at this stage	21
Occupational status (ISEI)	Excluded at this stage	860
Household registration	Excluded at this stage	73
Log GDP per capita	Excluded at this stage	195
Party membership	Excluded at this stage	12
Final analytical sample	Complete model variables and positive weights	6,871

Notes: The model file contained 8,040 rows, including 8,038 individual records and two district/county-only placeholder rows, which were excluded. Of the original 8,148 survey respondents, 110 were already absent before this screening; reasons for that earlier loss could not be established from the retained processing records. Missingness across variables can overlap; the exclusion counts are sequential. Other model variables caused no further exclusions in this sequence. The final sample retains 80 respondents from one district/county with no green-facility records.

Medical-bed values had been imputed before complete-case selection for 29 districts/counties, covering 2,458 final-sample respondents. The value used was the median among districts/counties with observed data in the same province; where none was available, the median among all 55 districts/counties with observed values was used.

Supplementary Table S3. OLS models adjusted for population and administrative area

Variable	Model 1	Model 2	Model 3	Model 4
Green facility supply (log-transformed, standardized)	−0.037 (0.034)	−0.029 (0.031)	−0.041 (0.033)	−0.034 (0.030)
Spatial balance	0.046 (0.034)	0.014 (0.024)	0.058 (0.035)	0.027 (0.026)
Public transport connectivity	−0.112* (0.052)	−0.074 (0.051)	−0.104* (0.052)	−0.071 (0.051)
Occupational status (ISEI)	0.082*** (0.019)	0.082*** (0.019)	0.075*** (0.020)	0.076*** (0.019)
Spatial balance × public transport	—	−0.072** (0.022)	—	−0.065** (0.023)
Spatial balance × ISEI	—	—	−0.053** (0.019)	−0.047* (0.019)
Log resident population	0.060 (0.039)	0.074+ (0.038)	0.060 (0.039)	0.072+ (0.038)
Log administrative area	−0.111* (0.047)	−0.123** (0.047)	−0.106* (0.046)	−0.118* (0.046)
Individual-level controls	Yes	Yes	Yes	Yes
Other district/county-level controls	Yes	Yes	Yes	Yes
N	6,871	6,871	6,871	6,871
Districts/counties	82	82	82	82
R^2	0.144	0.147	0.146	0.148

Notes: District/county-clustered robust standard errors are in parentheses. + $p < 0.10$, * $p < 0.05$, ** $p < 0.01$, *** $p < 0.001$. All models use the model-file weight field as a probability weight (Stata: [pw=weight]) and cluster standard errors by county_id across 82 districts/counties, with 81 degrees of freedom for t tests.

All models adjust simultaneously for log resident population and log administrative area, together with age, sex, years of education, household registration, marital status, party membership, ethnicity, log GDP per capita, and log medical beds per 10,000 population. The supply measure is ln(1 + green facility POIs per 10,000 population), standardized before regression. Supply, spatial balance, public transport connectivity, and ISEI use the same standardization parameters, weights, and sample as Table 2.

Models 2 and 3 add the public transport and occupational-status interactions, respectively; Model 4 includes both. In interaction models, the spatial-balance main term is the conditional association when the included moderator or moderators equal zero on their standardized scales.
